# Hepatitis G virus infection in Egyptian children with chronic renal failure (single centre study)

**DOI:** 10.1186/1476-0711-8-36

**Published:** 2009-12-16

**Authors:** Ayman Mohammad Hammad, Mohammad Hosam El Deen Zaghloul

**Affiliations:** 1 Department of Pediatrics, Faculty of Medicine, Mansoura University, Mansoura, Egypt; 2 Department of Clinical Pathology, Faculty of Medicine, Mansoura University, Mansoura, Egypt

## Abstract

**Background:**

Hepatitis G virus (HGV) is an RNA virus. It is mainly transmitted through exposure to contaminated blood although other routes may also exist. Patients with chronic renal failure (CRF) are at high risk of acquiring HGV because they require frequent blood transfusions. Ongoing HGV infection can be only diagnosed by demonstrating viremia in patient sample by reverse transcriptase (RT) PCR. Antibodies to the envelop protein E_2 _(anti E_2_) of HGV is an indicator of virus clearance and testify past HGV contact. This cross sectional study was done to assess the frequency of HGV exposure (ongoing and past infection) in Egyptian children with CRF and to study the possible risk factors of infection.

**Methods:**

This study included 100 children with CRF [34 on regular haemodialysis (HD) and 66 before the start of dialysis (predialysis)]. All patients sera were tested for HGV RNA by RT-PCR, anti E_2_, hepatitis C virus (HCV) antibody, hepatitis B surface antigen (HBsAg), and hepatitis B core antibody (HBcAB). Twenty five healthy children of matched age & sex were used as controls.

**Results:**

HGV RNA was positive in 9 (26.5%) of HD and 9 (13.6%) of predialysis children. Anti E_2 _was positive in 14 (41.2%) of HD and 19 (28.8%) of predialysis children.

In comparison to controls; CRF (n = 100); HD and predialysis children had significantly higher prevalence of anti E_2 _[4% VS 33% for all CRF cases; (p = 0.002)& 41.2% (p = 0.002) and 28.8% (p = 0.01); for HD and predialysis groups; respectively]. HGV RNA was significantly more prevalent only in HD children in comparison to controls (p = 0.03). HD and predialysis children did not have significant difference in the prevalence of HGV RNA (p = 0.16) or anti E_2 _(p = 0.26).

HGV exposure was not correlated with positivity of anti HCV (p = 0.32), HCV RNA (0.09), HBsAg/HBcAB (p = 1), age (p = 0.06), or gender (p = 0.83). It was significantly correlated with duration of the disease (p < 0.001). Ongoing HGV infection was significantly more prevalent with frequent blood transfusion (p < 0.001). There were no significant differences in serum levels of ALT (p = 0.09), total bilirubin (p = 0.1) and albumin (p = 0.06) in children with ongoing infection in comparison to healthy controls.

**Conclusions:**

The frequency of HGV exposure in Egyptian children with CRF appears to be high and is mainly related to frequent blood transfusions and longer disease duration. HGV infection in these children is not associated with significant changes in hepatic biochemical parameters.

## Background

Infections are one of the important causes of morbidity and mortality in patients with end stage renal failure[1]. Chronic hepatitis is a major complication of chronic haemodialysis (HD). Initially; hepatitis B virus (HBV) infection was the most common etiologic agent of chronic hepatitis in patients on chronic HD. Later; after HBV vaccines became available and measures for screening and exclusion of hepatitis B surface antigen (HBsAg) positive blood were routinely used, HBV infection dropped significantly. Subsequently; however, hepatitis C virus (HCV) emerged as a new problem. In USA; rates of positive anti HCV reached up to 36% in HD patients in 1990s [2].

Two different laboratories in the USA isolated a new flavivirus-like RNA virus in the years 1995 and 1996. The first laboratory named it "G B virus-C (GBV-C)" and the other "hepatitis G virus (HGV)". Both viruses were subsequently considered different genotypes of the same virus because they were found to share most of the nucleotide and amino acid sequences [3]. Both viruses have a single stranded RNA genome of approximately 9.4 kb. It encodes a single poly protein of 2900 amino acids in which the non structural proteins are located at the C terminal end and the structural proteins at the N terminal end [4]. The HGV genome encodes an open reading frame coding for two envelop proteins (E1 & E2) [5].

The ongoing HGV infection can be diagnosed by demonstration of viremia in patient blood by reverse transcriptase (RT)-PCR. An assay detecting antibodies to the envelop protein E2 (anti E2) of HGV has been developed and this serological marker is considered to be an indicator of the virus clearance [6,7]. Thus; the presence of anti E2 seems to indicate past HGV exposure and is associated with immunity and protection from reinfection [8].

Blood transfusion is the main risk factor for HGV transmission [9]. Patients with chronic renal failure (CRF) usually require frequent blood transfusions which make them more vulnerable to HGV infection [10,11]. The present data on the prevalence of HGV anti E2 in HD patients is conflicting, with studies showing rates of 7% in Japan [12] up to 29% in Germany [13]. Up to our knowledge there are no published data on the prevalence of HGV infection in Egyptian children with CRF.

HGV associated hepatitis runs with normal biochemical parameters in 75% of patients [14]. Although HGV infection appears to be not associated with severe hepatic damage; however a possible link between HGV infection and acute [15-17], fulminant hepatitis [18-21], chronic (mild and moderate) hepatitis [22,23] or even hepatic fibrosis [24] has been suggested. This study was done to assess the frequency of HGV exposure (ongoing & past infection) in Egyptian children with CRF and to correlate viral exposure with age, sex, duration of disease, frequency of blood transfusion, and co infection with HBV & HCV and to study the effect of ongoing viral infection on hepatic biochemical parameters in these children.

## Methods

This cross sectional study was carried out at Mansoura University Children's Hospital Nephrology Unit, Egypt in the period from January till June 2008. The study involved 100 children (67 males & 33 females) with CRF. Their ages ranged from 2 to 18 years [median (Interquartile range; IQR) = 9 (5-14) years]. Written concents were obtained from the parents of children and the study was approved by the ethical committee of the university.

Among the studied patients; 34 children (19 males & 15 females) were on regular HD for a mean duration of 5.1 ± 2.1 years and the remaining 66 children (48 males & 18 females) were on medical therapy without a need to receive dialysis with mean disease duration of 2.4 ± 0.94 years.

A questionnaire was used to collect sociodemographic data such as age, sex, number of previous blood transfusions and duration of the disease. A group of 25 randomly chosen apparently healthy children of matched age and sex served as a control group.

Five mls. of blood samples were withdrawn from patients and controls. They were centrifuged and serum was separated. Serum was stored in aliquots at -70°C. Repeated freezing and thawing was avoided. All the biochemical parameters were done by routine laboratory methods unless otherwise mentioned. HBsAg, Total hepatitis B core antibody (HBcAB) and Anti HCV antibody were detected with commercially available enzyme linked immunosorbent assay (Equipar, 21047 Saronno VA, Italy). Serum alanine aminotransferase (ALT) values were measured by Hitachi 902 auto analyzer (Roche, Switzerland). HGV Anti E2 was detected by ELISA and HGV RNA by RT - PCR.

### HBsAg

This kit uses double antibody (sandwich) ELISA method, in which polystyrene microwell strips are pre-coated with antibody to HBsAg. Patients samples are added to the microwells together with a second antibody; horseradish peroxidase (HRP-Conjugate). During incubation the specific immunocomplex formed in case of presence of HBsAg in the sample, is captured on the solid phase. After washing, chromogen solution containing Tetramethylbenzidine (TMB) is added to the wells in presence of the antibody -antigen -antibody (HRP) immunocomplex, the colorless chromogens are hydrolyzed by the bound HRP conjugate to a blue colored product. The blue color turns yellow after the reaction is stopped with sulfuric acid. The intensity of color is proportional to the amount of antigen captured in the wells, and to the sample respectively.

### HbcAb

HBcAb test uses the principle of competitive immunoassay technique for the detection of antibodies to HBc in human serum. During the assay, the test specimen and HRP-HBcAb conjugates are incubated simultaneously with the coated microwells. HBcAb if present in the specimen will compete with HRP-HBcAb for the constant amount of Hbc antigen coated on the microwells surface. Thus the amount of HRP-HBcAb bound to the well is inversely proportion to the concentration of HBcAb in the specimen. Unbounded conjugated are then removed by washing. The presence of the HRP-complex is shown by a blue color upon additional incubation with TMB substrate.

### HCV-Ab

This kit employs solid phase ELISA method for detection of antibodies to HCV in two step incubation procedure. Polystyrene microwell strips are precoated with recombinant antigens corresponding to the core and the non-structural regions of HCV. During the first incubation step, specific HCV-Ab, if present, are captured on the solid phase. The wells are washed and anti-human igG antibodies conjugated to the enzyme (HRP - Conjugate) are added. During the second incubation step, these HRP Conjugate antibodies bind to any antigen antibody complexes previously formed and the unbound HRP Conjugate is then removed by washing. Chromogen solution containing TMB is added to the wells in presence of the antigen-antibody-anti-IgG (HRP) immunoconplex which is hydrolyzed to a blue colored product. The blue color turns yellow after the reaction is stopped with sulfuric acid. The intensity of color can be measured and it is proportional to the amount of antibody captured in the wells, and to the amount of antibody in the sample respectively.

### Hitachi 902

A random access fully automated chemistry analyzer with a throughput up 200 tests per hour and up to 300 tests with optional ion selective electrode. The Roche/Hitachi 902 test menu comprises clinical chemistry, protein profiling and therapeutic drug monitoring. It is able to consolidate routine testing and support specials testing assays and a choice of over 100 different assay applications.

### HGV Anti E2 Testing

All of the samples were tested with a commercial Enzyme-linked Immunosorbent Assay (Anti-H Genv EIA; Boehringer, GmbH, Mannheim, Germany) for the detection of specific IgG antibodies against HGV E2 protein. Antibodies to the E2-protein of HGV were detected by a two-step sandwich enzyme immunoassay [7]. Serum samples or controls were diluted in sample buffer and were added to coated microtiter wells. The microplate was incubated and washed then a solution containing peroxidase-conjugated anti-human IgG antibody was added. After incubation at room temperature and the addition of chromogen substrate, the optical density was measured at 405 nm within 10 minutes. All the incubation steps were performed using a shaker. Results were interpreted according to the manufacturer's instruction.

### RT-PCR for HGV RNA

Total RNA was extracted from 100 μL of serum using an RNA easy Mini kit (Qiagen, Valencia, California, USA). The isolated RNA was used for reverse transcription (RT) and first round PCR. RT-PCR was performed in a single tube using RT-PCR System (Promega, Madison, WI). Both first and second-round PCR were carried out using primers that hybridize to 5' non-translated regions of an infectious GBV-C clone (GenBank accession no. AF121950, nt 54 to 389). Primers for the first-round RT-PCR were GBVF1 5'-CCGACGCCTATCTAAGTA GACGC and GBVR1 5'-TCAACTCGCCGGATAAA-CCTATTGG. Primers for the second-round PCR were GBVF2 5'-GTGACAGGGTT-GTAGG and GBVR2 5'-GACATTGAA-GGG-CGACGTGG. PCR products were detected on 1.5% agarose gels containing 0.5 μg/ml ethidium bromide. The expected band sizes were 336 and 231 bp for the first- and second-round PCR; respectively. A reaction was considered positive if either the first- or second-round PCR produced a band of the expected size [25].

### Qualitative HCV RNA detection (Cobas Amplicor, Roche Diagnostics)

The amplicor HCV RNA detection is based on five major processes; specimen preparation, reverse transcriptase of target RNA to generate complementary DNA, PCR amplification of target DNA using HCV complementary primers, hybridization of the amplified products to oligonucleotide probes specific to the targets and detection of the probe - bound amplified products by colorimetric determination. The master mix reagent has been developed to insure equivalent detection of all known genotypes of HCV at concentration of 50 Iu/ml.

### Statistical analysis

Data was analyzed by SPSS (Statistical Package for Social Sciences) under Windows (version 10). Numerical data were found to have a non parametric distribution by Kolmogorov Smirnov test. Data were expressed as median and interquartile range (IQR) or when indicated as absolute numbers and percentages. Tests used included Chi square and Mann Whitney test. P value less than 0.05 was considered significant.

## Results

Table 1 gives the frequency of HGV, HCV and HBV markers in the two groups of patients and controls. Among the 100 CRF patients; HGV exposure (ongoing and past infection) was found in (51\100) 51% of children. HGV RNA and Anti E2 were not detected simultaneously in any patient. Among the HD group: HGV RNA, anti E2 antibodies, HBsAg; HbcAB, HCV RNA and anti HCV were positive in (9/34) 26.5%; (14/34) 41.2%; (2/34) 5.9%; (2/34) 5.9%; (33/34) 97% and (32/34) 94.1% of patients; respectively.

**Table 1:** Frequency of HGV RNA, Anti E2, HBsAg, HbcAB, HCV RNA and Anti HCV in patients and controls.

	*HGV Exposure*	*HGV/RNA*	*Anti E2*	*HbsAg*	*HBcAB*	*HCV RNA*	*Anti HCV*
**Haemodialysis(34)** **Predialysis (66)**	**23(67.6%)** **28(42.4%)**	**9 (26.5%)** **9 (13.6%)**	**14 (41.2%)** **19 (28.8%)**	**2 (5.9%)** **3 (4.5%)**	**2 (5.9%)** **3 (4.5%)**	**33 (97%)** **25 (37.9%)**	**32 (94.1%)** **20 (30.3%)**
**Total (100)**	**51 (51%)**	**18 (18%)**	**33 (33%)**	**5 (5%)**	**5 (5%)**	**58 (58%)**	**52 (52%)**

**Controls (25)**	**2 (8%)**	**1 (4%)**	**1 (4%)**	**0**	**0**	**0**	**0**

In the predialysis group: (9/66) 13.6%; (19/66) 28.8%; (3/66) 4.5%; (3/66) 4.5%; (25/66) 37.9% and (20/66) 30.3% of patients tested positive for HGV RNA, Anti E2, HBsAg, HbcAB. HCV RNA and anti HCV; respectively.

HGV RNA was detected in significantly more children on HD (26.5%) than in controls (4%) (p = 0.03). On the other hand; in comparison to controls; all CRF, HD and predialysis children had significantly higher prevalence of anti E2 [4% vs. 33% for all CRF cases; (p = 0.002), 41.2% (p = 0.002) and 28.8% for HD and predialysis groups (p = 0.01); respectively].

HD and predialysis children did not have significant difference in the prevalence of HGV RNA (p = 0.16) or anti E2 (p = 0.26).

Demographic and virological markers were compared between children with HGV exposure (ongoing infection or past infection) and those who were not exposed. HGV exposure was not related to age (p = 0.06), sex (p = 0.83), history of blood transfusion (p = 0.15), co infection with HCV (p = 0.09) or HBV (p = 1). HGV exposed patients had significantly longer disease duration in comparison to none exposed patients (p < 0.001), (Table 2).

**Table 2:** Comparison of different parameters in patients with and without HGV exposure.

	*HGV Exposed*	*HGV None Exposed*	*P*
**Number of patients**	51	49	-
**Age (years)**	7 (5-13)	9 (7-15)	0.06*
**Sex (M : F)**	35 : 16	32 : 17	0.83**
**HBsAg (+)**	3	2	1.o**
**HCV RNA (+)**	26 (50.1%)	32 (65.3%)	0.09**
**History of blood** **transfusion (+)**	49 (96%)	43 (87.7%)	0.15**

**Duration (years)**	3 (3-5)	2 (2-3)	< 0.001*

Values expressed as percentages or median and IQR

* Mann Whitney test

**Chi Square test

The number of blood transfusions was studied in relation to ongoing HGV infection. Patients with ongoing infection had significantly more frequent blood transfusion in the last 12 months preceding sampling in comparison to patients without ongoing infection [median (Interquartile range; IQR) = 9 (7-24) Vs 2 (0-5) times; p < 0.001; respectively].

The significance of HGV viraemia in the liver biochemistry was also studied. There were no significant differences in serum levels of ALT (p = 0.09), total bilirubin (p = 0.1) and albumin (p = 0.06) in children with ongoing infection in comparison to healthy controls, (Table 3).

**Table 3:** Comparison of liver biochemistry in children with positive HGV RNA and controls.

	*Patients*	*Controls*	p*
**Albumin (Gm/dl)**	4 (3.8-4)	4 (3.9-4.2)	0.06
**Total bilirubin (mg/dl)**	0.9 (0.8-1)	0.8 (0.8-0.9)	0.1

**ALT (U/L)**	27 (21.7-33)	23 (20.5-28)	0.09

Values expressed as median and IQR

* Mann Whitney test

## Discussion

In the current work, the frequency of HGV exposure was (28/66) 42.4% in predialysis children with CRF and increased to (23/34) 67.6% in HD children. It was 51% (51/100) in the total group of CRF children and only 8% in healthy controls.

Patients with chronic renal failure have a great risk to acquire HGV infection because they usually need frequent blood transfusions [26]. HGV RNA prevalence in chronic dialysis adult patients has been studied in different countries with somehow conflicting results. HGV RNA prevalence rates in HD patients ranged from 11.5 to 27% in the USA [27,28], from 6-57.5% in Europe [11,16] and in Asia, from less than 2.2% in India [29] to 55% in Indonesia [30]. In contrast, few reports are available on HGV prevalence in children. HGV RNA is prevalent in 6.3% of healthy American children [31], only in 0.5% of healthy Japanese children [32], in 21% of Pakistani children with Beta thalassaemia [33], in 13% of Italian children with thalassaemia [34] and in 18.5% of American children with CRF [35]. As Egypt is one of the countries world wide suffering from high prevalence of hepatotropic viruses [36]; it is not surprising to find a high frequency of HGV exposure in the studied children.

Although a positive correlation between the prevalence of HGV infection and the history and/or amounts of blood transfusion have been reported in some studies [37,38]; other studies have found no such connections [16,39]. In our study; HGV exposure was not significantly different in children with and without history of blood transfusion; however patients with ongoing infection have significantly more frequent blood transfusions in the last 12 months preceding sampling in comparison to patients without ongoing infection. These apparently opposing findings can be explained easily if we took into consideration that a large number of our patients had history of blood transfusion. 49/51 (96%) of HGV exposed patients and 43/49 (87.7%) of HGV non exposed patients had history of blood transfusion. Our centre is a tertiary referral facility and most of our patients present to us during late stages of renal diseases after they have received blood transfusion for treatment of anemia of CRF in their primary health care facilities. Thus when we studied HGV exposure in relation to history of blood transfusion; we did not find significant difference between patients with and without history of blood transfusion. It is logic to think that every blood transfusion session carries a potential risk of getting blood borne infection and that the number of blood transfusions should be considered in evaluating the potential risk of getting any blood borne infection; so when we studied the relation between ongoing HGV infection and the number of blood transfusions in the last 12 months before sampling; we found that HGV infection was significantly more common with frequent transfusions.

Several other non parenteral routes of infection have been suggested for HGV infection such as percutaneous contamination from saliva [40], maternal-fetal vertical transmission [41] and nosocomial infection through patient to patient transmission [42]. In our study, HGV exposed patients had significantly longer disease duration in comparison to none exposed patients. We believe that longer disease duration could increase the chance to get HGV infection through exposure to increasing numbers of blood transfusion and/or more exposure to the above mentioned suggested non parenteral modes of infection.

Numerous authors have studied the association between HCV and HGV infection with variable results [39,43,44]. HGV co infection has been reported in up to 21% [45] of patients with chronic HCV infection. Fabrizi et al., reported that the frequency of anti HCV antibody was significantly higher in HGV positive than in HGV negative patients on chronic HD treatment and that the rate of co infection was 82% [46]. On the other hand, none of HGV positive HD patients were found to be co infected with HCV in an Italian [47] and Iranian study [48]. In our study the prevalence of HGV in the HCV RNA positive group was not significantly different than in the HCV RNA negative group. Therefore HGV and HCV appear to be transmitted independently as previously reported [47,48].

Some studies have reported coinfection between HGV and HBV in dialysis patients with rates varying from 2.3 to 13% in HD patients [39,49,50]. Other studies have not found any association between these viral infections [51]. In our study; only 5% of CRF children were positive for HBsAg and HBcAB. Again there was no significant difference in the prevalence of HGV infection between HBsAg/HBcAB positive and negative children.

The simultaneous detection of HGV RNA and anti E2 is uncommon and appears just before clearance, probably in the form of immunocomplex [52]. In the current work; none of the anti E2 positive patients tested positive for HGV RNA. Similarly none of the HGV RNA positive patients was serologically positive. This confirms that the appearance of the antibody is accompanied by clearance of HGV RNA. Similar findings have been previously reported [7,53].

In the current study, serum ALT, total bilirubin and albumin levels were not significantly elevated in children with ongoing infection in comparison to controls. Although children with ongoing infection had slightly elevated serum ALT levels in comparison to controls [median (IQR) 27 (21.7-33) Vs 23 (20.5-28) U/L; respectively); this was not statistically significant. HGV infection was reported to be associated with acute [15-17] up to fulminant hepatitis [18-21]. On the contrary; other researchers have suggested that in most instances HGV infection does not cause an elevation of ALT levels [37]. Histopathologic examination of liver biopsies revealed no differences between patients with HCV infection alone and patients who were co infected with HGV [54]. Pessoa et al; reported that the hepatic to serum median ratio of HGV RNA was less than 1, in contrast to HCV which is present in high ratio [55]. The current work suggests that HGV infection in children with CRF does not cause significant elevation in ALT levels and therefore there is no strong indication to consider HGV as an etiologic agent of symptomatic hepatitis in these children. Another possible explanation is the small number of children with ongoing infection in our study (18 patients) which may have affected the relevance of ALT values in comparison to controls. A study of larger number of children with ongoing HGV infection is required.

## Conclusion

In summary, the frequency of HGV exposure in Egyptian children with CRF appears to be high and is mainly related to frequent blood transfusions and longer disease duration. HGV infection in these children is not associated with significant changes in hepatic biochemical parameters.

## Competing interests

The authors declare that they have no competing interests.

## Authors' contributions

AMH was responsible for the choice of cases and the clinical data consultation, statistical analysis and drafted the manuscript. MHZ is the one who suggested the idea and participated in the design of the study, carried out the molecular biology study and helped to draft the manuscript. All authors read and approved the final manuscript

## References

[B1] Greco M Cristiano K Leozappa G Rapicetta M Rizzoni G Hepatitis C infection in children and adolescents on hemodialysis and after renal transplant Pediatr Nphrol 1993 7 424 7 10.1007/BF00857557 8398653

[B2] Miles A Friedman E Schrier RW, Goottschalk CW Center and home chronic hemodialysis: outcome and complication Disease of the kidney 1997 Little Brown, Boston 2833 44

[B3] Zuckerman A Alphabet of hepatitis viruses Lancet 1996 347 558 9 10.1016/S0140-6736(96)91267-2 8596314

[B4] Roth W Waschk D Marx S Tschauder S Zeuzem S Bialleck H Weber H Seifried E Prevalence of hepatitis G virus and its strain variant, the GB agent, in blood donations and their transmission to recipients Transfusion 1997 37 651 6 10.1046/j.1537-2995.1997.37697335162.x 9191828

[B5] Katayama K Kageyama T Fukushi S Hoshino F Kurihara C Ishiyama N Okamura H Oya A Full-length GBV-C/HGV genomes from nine Japanese isolate: characterization by comparative analyses Arch Virol 1998 143 1063 75 10.1007/s007050050356 9687865

[B6] Dille B Surowy T Gutierrez R Coleman P Knigge M Carrick R Aach R Hollinger F Stevens C Barbosa L Nemo G Mosley J Dawson G Mushahwar I An ELISA for detection of antibodies to the E2 protein of GB virus C J Infect Dis 1997 175 458 61 10.1093/infdis/175.2.458 9203673

[B7] Tacke M Kiyosawa K Stark K Schlueter V Ofenloch-Haehnle B Hess G gel A Detection of antibodies to a putative hepatitis G virus envelope protein Lencet 1997 349 318 20 10.1016/S0140-6736(96)06461-6 9024375

[B8] Thomas D Vlahov D Alter Hunt J Marshall R Astemborski J Nelson K Association of antibody to GB virus C (hepatitis G virus) with viral clearance and protection from reinfection J Infect Dis 1998 539 42 10.1086/514245 9498429

[B9] Alter H Nakatsuji Y Melpolder J Wages J Wesley R Shih J Kim J The incidence of transfusion-associated hepatitis G virus infection and its relation to liver disease N Engl J Med 1997 747 57 10.1056/NEJM199703133361102 9052652

[B10] Masuko K Mitsui T Iwano K Yamazaki C Okuda K Meguro T Murayama N Inoue T Tsuda F Okamoto H Miyakawa Y Mayumi M Infection with hepatitis GB virus C in patients on maintenance hemodialysis N Engl J Med 1996 1485 90 10.1056/NEJM199606063342301 8618602

[B11] de Lamballerie X Charrel R Dussol B Hepatitis GB virus C in patients on hemodialysis N Engl J Med 1996 1549 54 10.1056/NEJM199606063342319 8618624

[B12] Shibuya A Takeuchi A Kamata K Saigenji K Kobayashi N Yoshida A Prevalence of hepatitis G virus RNA and anti-E2 in a Japanese haemodialysis population Nephrol Dial Transplant 1998 2033 36 10.1093/ndt/13.8.2033 9719160

[B13] Schulte-Frohlinde E Schmolke S Reindl W Schätzle G Scherf J Kopp K Classen M Schlüter V Significance of antibodies to recombinant E2 protein of hepatitis G virus in haemodialysis patients J Viral Hepat 1998 5 341 4 10.1046/j.1365-2893.1998.00122.x 9795918

[B14] Arican A Sengezer T Bozdayi M Bozkaya H Ucgul E Dincol D Uzunalimoglu O Prevalence of hepatitis-G virus and hepatitis-C virus infection in patients with non-Hodgkin's lymphoma Med Oncol 2000 17 123 6 10.1007/BF02796207 10871818

[B15] Ling B Zhuang H Cui Y An W Li Z Wang S Zhu W A cross-sectional study on HGV infection in a rural population World J Gastroenterol 1998 4 489 92 11819351 10.3748/wjg.v4.i6.489 PMC4723435

[B16] Corun C Jadoul M Loute G Goubau P Hepatitis G virus infection in haemodialysis patients: epidemiology and clinical relevance Nephrol Dial Transplant 1997 12 1326 9 10.1093/ndt/12.7.1326 9249765

[B17] Yashina T Favorov M Khudyakov Y Fields H Znoiko O Shkurko T Bonafonte T Sevall J Agopian M Peter J Detection of hepatitis G virus (HGV) RNA: clinical characteristics of acute HGV infection J Infect Dis 1997 175 1302 7 10.1086/516460 9180167

[B18] Moaven L Locarnini S Bowden D Kim J Breschkin A McCaw R Yun A Wages J Jr Jones B Angus P Hepatitis G virus and fulminant hepatic failure: Evidence for transfusion related infection J Hepatol 1997 27 613 9 10.1016/S0168-8278(97)80077-3 9365036

[B19] Sheng L Soumillion A Beckers N Wu C Verslype C Nevens F Pirenne J Aerts R Kosala H Fevery J Yap S Hepatitis G virus infection in acute fulminant hepatitis: prevalence of HGV infection and sequence analysis of a specific viral strain J Viral Hepat 1998 5 301 6 10.1046/j.1365-2893.1998.00123.x 9795913

[B20] Yoshiba M Okamoto H Mishiro S Detection of the GBV-C hepatitis virus genome in serum from patients with fulminant hepatitis of unknown etiology Lancet 1995 346 1131 2 10.1016/S0140-6736(95)91802-7 7475605

[B21] Pessoa M Wright T Hepatitis G virus: what is the next step? Liver Transpl Surg 1997 3 677 9 10.1002/lt.500030625 9404976

[B22] Rey D Vidinic-Moularde J Meyer P Schmitt C Fritsch S Lang J Stoll-Keller F High prevalence of GB virus C/hepatitis G virus RNA and antibodies in patients infected with human immunodeficiency virus type 1 Eur J Clin Microbiol Infect Dis 2000 19 721 4 10.1007/s100960000352 11057510

[B23] Di Bisceglie A Hepatitis G virus infection: a work in progress Ann Intern Med 1996 125 772 3 8929013 10.7326/0003-4819-125-9-199611010-00013

[B24] Ilchenko L Yu Sharafanova T Shepeleva S Serova T Antibodies to hepatitis G virus in patients with chronic liver diseases Hepatology 2003 5 4 6

[B25] Smith S Donio M Singh M Fallon J Jitendranath L Chkrebtii N Slim J Finkel D Perez G Prevalence of GB virus type C in urban Americans infected with human immunodeficiency virus type 1 Retrovirology 2005 2 38 43 10.1186/1742-4690-2-38 15927079 PMC1175858

[B26] Sheng L Widyastuti A Kosala H Donck J Vanrenterghem Y Setijoso E Soumillion A Verslype C Schelstraete R Emonds M Hess G Yap S High prevalence of hepatitis G virus infection compared with hepatitis C virus infection in patients undergoing chronic hemodialysis Am J Kidney Dis 1998 31 366 8 10.1053/ajkd.1998.v31.pm9469490 9469490

[B27] Bastani B Frenche D Gellens M Di Bisceglie A infection with hepatitis G (HGV) and hepatitis C (HCV) in patients on chronic haemodialysis Am J Nephrol 1996 9 199A

[B28] de Medina M Ashby M Schlüter V Hill M Leclerq B Pennell J Jeffers L Reddy K Schiff E Hess G Perez G Prevalence of hepatitis C and G virus infection in chronic hemodialysis patients Am J Kid Dis 1998 31 224 6 10.1053/ajkd.1998.v31.pm9469491 9469491

[B29] Desai M Pal R Banker D GB virus C/hepatitis G virus infection in Indian blood donor and high-risk groups Transfus Apher Sci 2004 30 111 7 10.1016/j.transci.2003.10.006 15062748

[B30] Tsuda F Hadiwandowo S Sawada N Fukuda M Tanaka T Okamoto H Miyakawa Y Mayumi M Infection with GB virus C (GBV-C) in patients with chronic liver disease or on maintenance hemodialysis in Indonesia J Med Virol 1996 49 248 52 10.1002/(SICI)1096-9071(199607)49:3<248::AID-JMV15>3.0.CO;2-8 8818973

[B31] Handa A Jurban R Dickstein B Boylan A Luban N Young N Brown K GB virus C/Hepatitis G virus infection is frequent in American children and young adults Clin Infect Dis 2000 30 569 71 10.1086/313702 10722444

[B32] Mizutani F Sugiyama K Goto K Ando T Terabe K Wada Y The prevalence of serum GB virus C/hepatitis G virus RNA and anti-E2 in Japanese children without a history of blood transfusion Tohoku J Exp Med 2000 190 185 92 10.1620/tjem.190.185 10778802

[B33] Moatter T Adil S Haroon S Azeemuddin S Hassan F Khurshid M Prevalence of hepatitis G virus in Pakistani children with transfusion dependent beta thalassemia major Indian J Pathol Microbiol 1999 42 475 82 11127381

[B34] Kondili L Chionne P Dettori S Badolato M Grosso A Vania A Rapicetta M GB virus C/Hepatitis G Virus Exposure in Italian Pediatric and Young Adult Thalassemic Patients Infection 2001 219 21 5 10.1007/s15010-001-9172-7 11545484

[B35] Szabo A Sallay P Kribben A Ross S Roggendorf M Tulassay T Philip T Heemann U Hepatitis G Virus Infection in Children on Dialysis and After Renal Transplantation Pediatr Nephrol 1998 12 93 5 10.1007/s004670050411 9543362

[B36] Habib M Mohamed M Abdel-Aziz F Magder L Abdel-Hamid M Gamil F Madkour Mikhail N Anwar W Strickland G Fix A Sallam I Hepatitis C virus infection in a community in the Nile Delta: risk factors for seropositivity Hepatology 2001 33 248 53 10.1053/jhep.2001.20797 11124843

[B37] Watanabe T Ishiguro M Kametani M Sugai Y Takakuwa K Akahane Y Massuko K Shimizu M Kojima M Fujita K Tsuda F Okamoto H GB virus C and hepatitis C virus infection in hemodialysis patients in eight Japanese centers Nephron 1997 76 171 5 10.1159/000190165 9200408

[B38] Campo N Sinelli N Brizzolara R Torre F Gurreri G Russo R Saffioti S Cello G Picciotto A Hepatitis G virus infection in hemodialysis and in peritoneal dialysis patients Nephron 1999 82 17 21 10.1159/000045362 10224479

[B39] Forns X Fernandez-Llama P Costa J Lopez-Labrador F Ampursanes S Olmedo E Saiz J Guilera M Lopez-Pedret J Sanchez-Tapias J Darnell A Jimenez de Anta M Ordinas Rodes J Hepatitis G virus infection in a hemodialysis unit: prevalence and clinical implication Nephrology, Dialysis, Transplantation 1997 12 956 60 10.1093/ndt/12.5.956 9175049

[B40] Ustjindao Y Hizel N Boyacioolu S Akalin E Detection of hepatitis GB virus-C and HGV genomes in the saliva of patients undergoing maintenance hemodialysis Nephrology, Dialysis, Transplantation 1997 12 2807 10.1093/ndt/12.12.2807a 9430914

[B41] Lin H Kao J Yeh K Liu D Chang M Chen P Chen D Mother-to-infant transmission of GB virus-C/hepatitis G virus: the role of high-titered maternal viremia and mode of delivery Journal of Infectious Diseases 1998 177 1202 6 10.1086/515264 9593004

[B42] Liyu H Cornu C Jadoul M Dahan K Loute G Goubau P Molecular analysis of GB virus C isolates in Belgian hemodialysis patients Journal of Medical Virology 1998 55 118 22 10.1002/(SICI)1096-9071(199806)55:2<118::AID-JMV6>3.0.CO;2-5 9598931

[B43] Stransky J The discovery of hepatitis G virus J Czech Physicians 1996 4 99 101 8625384

[B44] Wang Y Chen H Fan M Liu H An P Sawada N Tanaka T Tsuda F Okamoto H Infection with GB virus C and hepatitis C virus in hemodialysis patients and blood donors in Beijing J Med Virol 1997 52 26 30 10.1002/(SICI)1096-9071(199705)52:1<26::AID-JMV5>3.0.CO;2-T 9131454

[B45] Martinot M Marcellin P Boyer N Detmer J Pouteau M Castelnau C Degott C Aupérin A Collins M Kolberg J Wilber J Benhamou J Erlinger S Influence of hepatitis G virus infection on the severity of liver disease and response to inferno alpha in patients with chronic hepatitis C Ann Int Med 1997 126 874 81 9163288 10.7326/0003-4819-126-11-199706010-00004

[B46] Fabrizi F Lunghi G Bacchini G Corti M Guarnori I Raffaele L Erba G Pagano A Locatelli F Hepatitis G virus infection in chronic dialysis patients and kidney transplant recipients Nephrol Dial Transplant 1997 12 1645 51 10.1093/ndt/12.8.1645 9269643

[B47] Tringali G Prevalence of HGV infection in hemodialysis patients from eastern Sicily Giorn Ital Nefrol 1998 15 141 7

[B48] Eslamifar A Hamker R Ramezani A Ahmadi F Gachker L Jalivand S Adibi L Atabak S Khameneh A Ghadimi R Aghakhani A Hepatitis G Virus Exposure in Dialysis Patients Int Urol Nephrol 2007 39 1257 63 10.1007/s11255-007-9267-x 17786579

[B49] Klusonova H Pliskova L Palicka V Fixa P Prevalence of viral hepatitis G infection in hemodialysis patients and coinfection with viral hepatitis B and C Vnitr Lek 2001 47 678 81 11789005

[B50] López-Alcorocho J Barril G Ortiz-Movilla N Traver J Bartolomé J Sanz P Selgas R Carreño V Prevalence of Hepatitis B, hepatitis C, GB virus C/hepatitis G and TT viruses in predialysis and hemodialysis patients J Med Virol 2001 63 103 7 10.1002/1096-9071(20000201)63:2<103::AID-JMV1003>3.0.CO;2-E 11170045

[B51] Fabrizi F De Vecchi A Lunghi G Finazzi S Bisegna S Ponticelli C Epidemiology of GB virus C/Hepatitis G virus infection in patients on peritoneal dialysis Perit Dial Int 2002 22 405 10 12227401

[B52] Mushahwar I Zuckerman J Clinical implications of GB virus-C J Med Virol 1998 56 1 3 10.1002/(SICI)1096-9071(199809)56:1<1::AID-JMV1>3.0.CO;2-3 9700625

[B53] Desassis J Laperche S Girault A Kolko A Bouchardeau F Zins B Poignet J Couroucé A Prevalence of present and past hepatitis G virus infection in a French hemodialysis centre Nephrol Dial Transplant 1999 14 2692 7 10.1093/ndt/14.11.2692 10534514

[B54] Bralet M Roudout-Thoraval F Pawlotsky J Bastie A Tran Van Nhieu J Duval J Dhemeaux D Zafrani E Histologic impact of GB virus C infection of chronic hepatitis Gastroenterology 1997 112 188 92 10.1016/S0016-5085(97)70234-8 8978358

[B55] Pessoa M Terrault N Detmer J Kolberg J Collins M Hassoba H Wright T Quantitation of hepatitis G and C viruses in the liver: evidence that hepatitis G virus is not hepatropic Hepatology 1998 27 877 80 10.1002/hep.510270335 9500722

